# Laser speckle contrast imaging system using nanosecond pulse laser source

**DOI:** 10.1117/1.JBO.25.5.056005

**Published:** 2020-05-25

**Authors:** Yuemei Zhao, Kang Wang, Weitao Li, Huan Zhang, Zhiyu Qian, Yangyang Liu

**Affiliations:** a Nanjing University of Aeronautics and Astronautics, Department of Biomedical Engineering, Nanjing, China; b Nanjing Institute of Technology, Nanjing, China

**Keywords:** cerebral blood flow, continuous wave, laser speckle contrast imaging

## Abstract

**Significance:** Nanosecond-pulsed laser has proven to be used to obtain the velocity of blood using the speckle contrast method. Without the scanning time, it has potential for achieving fast two-dimensional blood flow images in a photoacoustic imaging system with the same pulsed laser.

**Aim:** Our study aimed to evaluate the qualities of regional cerebral blood flow (rCBF) obtained in a laser speckle contrast imaging (LSCI) system using continuous wave (cw) and nanosecond pulse laser sources.

**Approach:** First, a LSCI system consisting of a cw laser with a wavelength of 632.8 nm and a cw laser/nanosecond pulse laser with a wavelength of 532 nm was developed. This system was used to obtain rCBF images of mouse *in vivo* with two different laser sources.

**Results:** Continuous wave lasers (532 and 632.8 nm) show different imaging characteristics for rCBF imaging. The rCBF images obtained using 532-nm nanosecond pulse laser showed higher resolution than those using 532-nm cw laser. There was no significant difference in the results using nanosecond pulse laser among various pulse widths or repetition rates.

**Conclusions:** It is proved that a nanosecond pulse laser could be used for LSCI.

## 1 Introduction

Many diseases, such as cardiovascular diseases, atherosclerosis, diabetes, and chronic venous insufficiency, cause functional and morphological changes in blood flow.^1^^,^^2^ The dynamic monitoring of blood flow has great value in life science research, drug evaluation, clinical diagnosis, clinical application, and surgical guidance. Currently, some effective measurement methods for living animal tissues, especially blood vessels, are being studied, such as magnetic resonance perfusion imaging, positron emission tomography (PET), x-ray angiography, fluorescence angiography, and laser Doppler flowmeter. However, there are some limitations in these blood flow imaging techniques.^3^^–^^5^ For example, magnetic resonance imaging (MRI) perfusion imaging and PET are mostly used for holistic imaging with low spatial and temporal resolution and high cost. Fluorescence angiography and x-ray angiography cannot provide functional information of blood flow and need injection of a contrast agent. Doppler flowmeter can only provide a single point of monitoring that does not provide a complete two-dimensional (2-D) map of blood flow velocity.^6^^–^^9^ Compared to other imaging techniques, laser speckle contrast imaging (LSCI) provides 2-D full-speed blood flow distribution with lower cost.

Moreover, photoacoustic imaging (PAI) using nanosecond pulsed laser has been widely used in high-sensitivity structural and blood flow. Yao and Wang^10^ measured transverse blood flow velocity based on photoacoustic Doppler bandwidth broadening. Chen et al.^11^ proposed flow speed *in vivo* measurement of capillaries by photoacoustic correlation spectroscopy. Liu et al.^12^ concluded that the rising time constant of the photoacoustic signals was linearly dependent on the flow speed. Liu et al.^13^ presented a fast optical-resolution photoacoustic microscopy to image blood flow speed with three-pulse excitation. However, it is time consuming for all these PAI methods to obtain 2-D images of blood flow, and multiple A-line imaging needs to be repeated at one spot.

Compared to a PAI system with nanosecond pulsed laser, the LSCI system with continuous wave (cw) laser provides 2-D full-speed blood flow distribution.^14^^–^^16^ In this study, we first proposed the application of the nanosecond pulse laser for measurement of regional cerebral blood flow (rCBF) in a LSCI system. Here, four blood flow imaging results irradiated by two different cw lasers (532 and 632.8 nm) and two 532-nm wavelength lasers (cw and nanosecond pulse) are analyzed. The performance of the 532-nm pulsed laser in LSCI is verified by experimental results for the first time. By applying a pseudocolor scale that corresponds to the contrast values of a speckle image, the blood flow speed distribution in the tissue can also be viewed. This study shows great significance in the formation of a multimode PAI system and the realization of an image fusion algorithm.

## 2 Methods

### 2.1 LSCI System with CW and Nanosecond Pulse Laser Sources

**Fig. 1:**
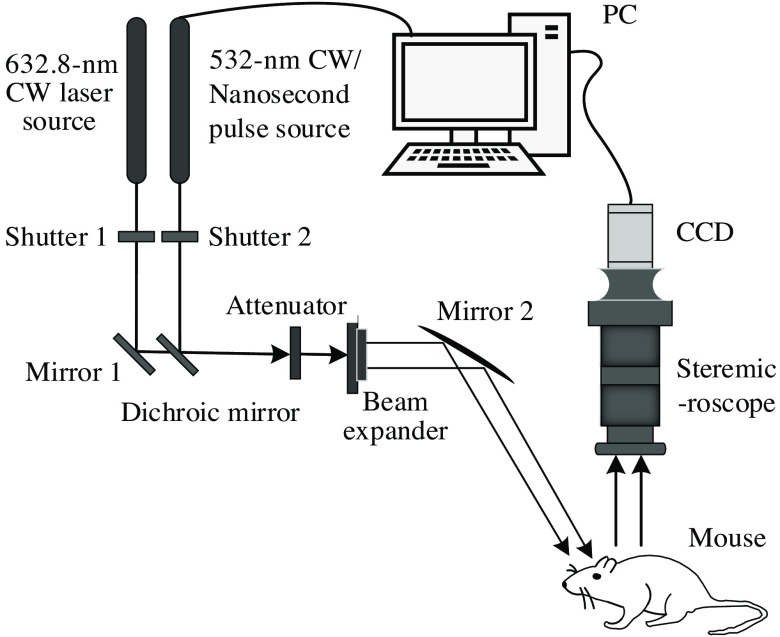
Schematic of the LSCI system with cw and nanosecond pulse laser sources.

The proposed system achieved imaging of blood flow under a combination of the 632.8- and 532-nm cw lasers as well as a 532-nm cw and pulse wave for the first time through optical path design, as shown in Fig. 1. The He–Ne laser (HNL150L-EC, Thorlabs) emits a 632.8-nm cw laser. The nanosecond pulse laser (VGEN-G-20, Spectra Physics) emits a 532-nm nanosecond pulse laser. High-performance green laser (MGL-III-532, CNI, China) emits a 532-nm cw laser. The 632.8-nm cw and 532.8-nm cw/nanosecond pulse lasers are controlled by shutters and reflected by mirrors and then pass through the dichroic mirror into the optical path system. The light then passes through an attenuator and is irradiated onto a beam expander (GBE15-A, Thorlabs), after which it reaches the brain window of the mouse. The brain window is a square of $6\times 6\;{\mathrm{mm}}^{2}$ in size, and the spot through beam expanding needs to meet the actual experimental requirements.

### 2.2 Experimental Process

Male Institute of Cancer Research mice (25 to 30 g) were purchased from the Animal Experiment Center. All animal experimental procedures were conducted in accordance with the guidelines under Institution Animal Care and Use Committee-approved protocols at Nanjing University of Aeronautics and Astronautics.

The mice were anesthetized with 5% chloral hydrate by intraperitoneal injection at 400  mg/kg (body weight). After the anesthesia was completed, the head and limbs of the mice were fixed on the stereotaxic brain locator in a recumbent position to facilitate craniotomy and reduce the noise caused by movement after the mice recovered. Because of the symmetry of the head of the mice, we drilled a rectangular area on one side (about 1 to 2.5 mm from the front halogen point and 1 to 4 mm from the middle line) to open the upper skull with a dental drill and use tweezers to open the meninges as the imaging area. Light reflected from the mouse is collected by an optical tube (12X Zoom, Navitar), collected by a 12-bit CCD camera (GS3-U3-51S5M-C, PointGrey, Canada), and stored in a computer. Considering the quality of image contrast and the velocity of blood flow, the exposure time of the CCD is chosen as 20 ms, and the blood flow images could be calculated using 30 frames of raw speckle images. Therefore, it would consume at least 600 ms to obtain one frame of blood flow image in each time point.

First, the cerebral vascular image of the mouse under white light was collected by CCD. Then the blood flow image of the mouse under the combination of a 632.8-nm cw laser with the 532-nm cw laser or with the 532-nm nanosecond pulse laser was obtained. After that, experimental data were collected and analyzed. The system achieves the combined measurements of 632.8- and 532-nm lasers blood flow as well as 532-nm cw and nanosecond pulse waves for the first time through optical path design.

### 2.3 Speckle Pattern Calculation

When a laser radiates on the cerebral cortex of the mice, the scattered light is collected by a CCD and raw speckle images will be formed. The motion of these scattered particles will lead to blurring in the raw speckle images. The level of blurring is quantified by the speckle contrast.^17^^,^^18^ This blurring degree is usually quantified by calculating speckle contrast K defined as

$$K=\frac{\sigma_{s}}{\left\langle I\right\rangle },$$ (1)

where $\sigma_{s}$ is the ratio of the standard deviation, I is the light intensity, and ⟨I⟩ is the mean light intensity. Considering the relation between the correlation time ($\tau_{c}$) and the exposure time (T) of the CCD, the speckle contrast K is given by

$$K=\frac{\sigma_{s}}{\left\langle I\right\rangle }={\left\{\frac{\tau_{c}}{2T}[1-\exp(\frac{-2T}{\tau_{c}})]\right\}}^{\frac{1}{2}}.$$ (2)

Then the relation between correlation time ($\tau_{c}$) and the decorrelation velocity v is defined as

$$\tau_{c}=\frac{1}{\alpha \times k_{0}\times v},$$ (3)

where α is determined by the Lorentz width and the liquid scattering characteristics. $k_{0}$ represents the wave velocity from which the velocity information (relative velocity) can be calculated. By analyzing the spatial statistical characteristics of the whole scattering pattern area, the velocity information of the corresponding particles can be obtained, and finally the particle velocity distribution map of the imaging area can be achieved.

### 2.4 SNR Calculation

When the experiment is completed, the signal-to-noise ratio (SNR) of the blood flow image obtained by the system is calculated, and the quality of the blood flow images under 532-nm cw and 532-nm nanosecond pulse lasers are compared. The method of calculating the SNR is: (1) select the region of interest (ROI) in the blood flow image and (2) obtain the mean and standard deviation of the ROI. The ratio of the mean and standard values is regarded as the SNR.

$$\mathrm{SNR}=\frac{{\text{Mean}}_{\mathrm{ROI}}}{{\text{Standard}}_{\mathrm{ROI}}}.$$ (4)

## 3 Results

### 3.1 Regional Cerebral Blood Flow Image with Different Wavelength CW Laser Source

**Fig. 2:**
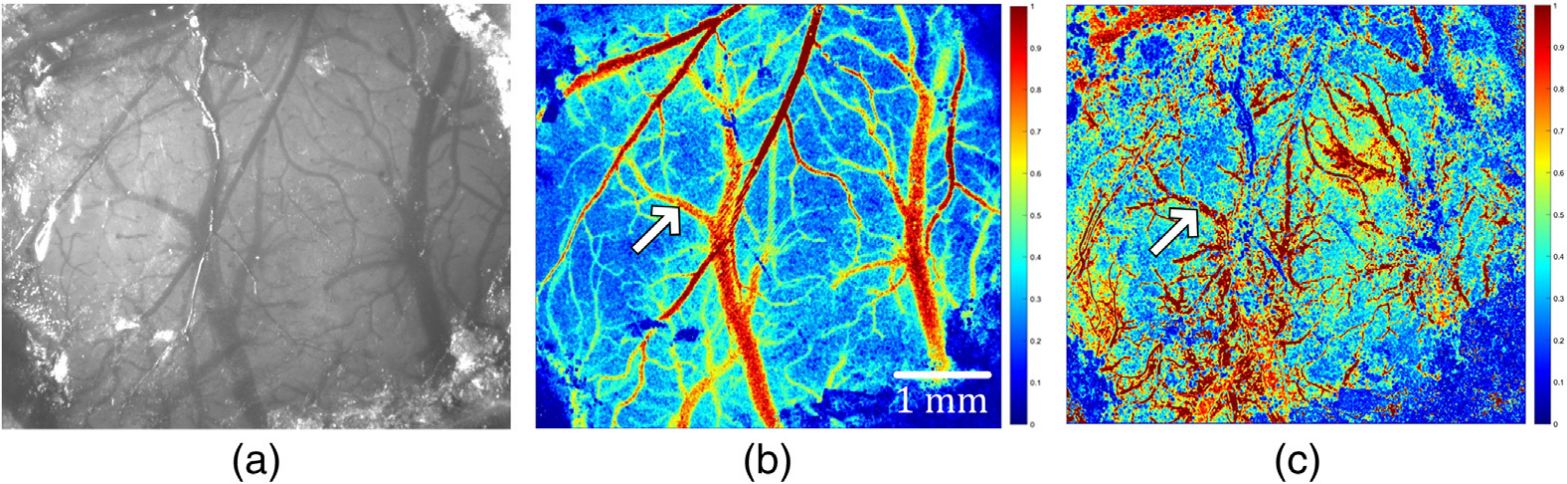
rCBF image with different wavelength cw laser sources: (a) white-light photo of blood vessels in the mouse brain, (b) rCBF image with 632.8-nm cw laser source, and (c) rCBF image with 532-nm cw laser source.

As shown in Fig. 2(a), the brain blood vessels of the mouse can be clearly seen under the white light. Under the irradiation of 632.8 nm cw laser, rCBF image of the thick blood vessels located at the surface of the mouse brain was shown in Fig. 2(b). However, Fig. 2(c) showed clear branches of the mouse brain and the trunk was clear. The blood vessels were not observable. At the same time, by comparing the images under white light, it was found that the 532-nm nanosecond pulse laser showed a good blood flow image on the blood vessels of the mouse brain at deeper depth. This experimental result obtained the brain blood flow images of mice at different wavelength lasers. According to the imaging characteristics of different wavelength lasers, an image fusion algorithm can be used to fuse the two images to obtain more abundant blood flow information.

### 3.2 Regional Cerebral Blood Flow Image with 532-nm Laser Source of Different Types

**Fig. 3:**
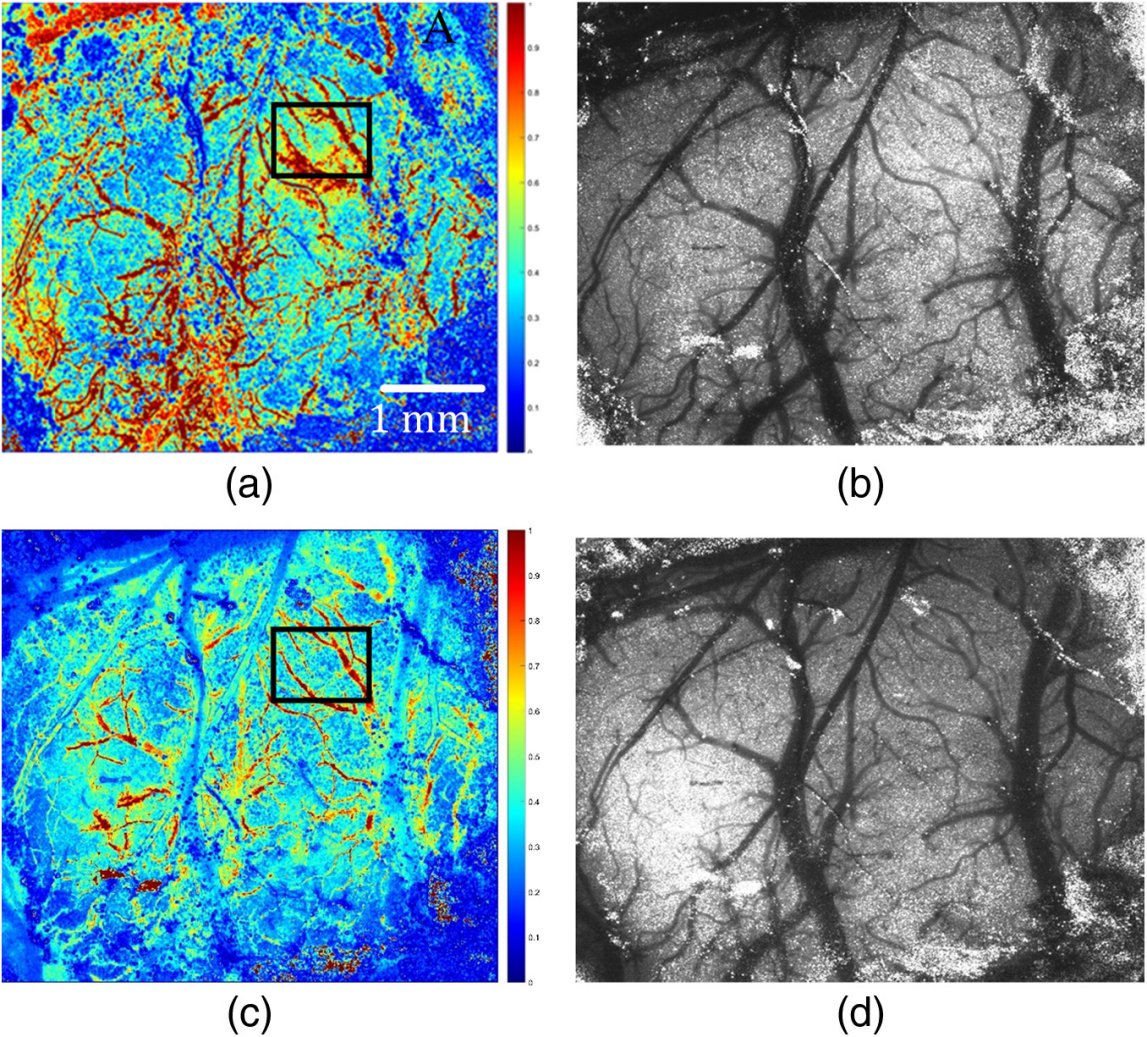
rCBF image with 532-nm laser source of different types: (a) cw laser source, (b) raw speckle images used to compute (a), (c) nanosecond pulse laser source (3 ns and 1200 kHz), and (d) raw speckle images used to compute (c).

Figure 3(c) shows the blood flow of equal thickness vessels in the main cerebral vessels of mice. Figure 3(a) shows the clear branches of the mouse brain and the clear trunk. In Figs. 3(b) and 3(d), the corresponding raw speckle images used to compute rCBF are provided. Comparing images of two modalities of the 532-nm laser, we found that the 532-nm nanosecond pulse laser exhibits blood flow change with higher resolution at a pulse width of 3 ns, capturing more effective blood vessel detailed information than the 532-nm cw laser (refer to Fig. 7 of the SNR results). The 532-nm cw laser showed more noise in the deep blood vessels of mouse brain. The research based on this image can only be carried out after denoising. According to the imaging characteristics of different types of laser sources, using an image fusion algorithm to fuse the two images will overcome their shortcomings.

### 3.3 Regional Cerebral Blood Flow Images of Different Repetition Rates with 532-nm Nanosecond Pulse Laser Source

**Fig. 4:**
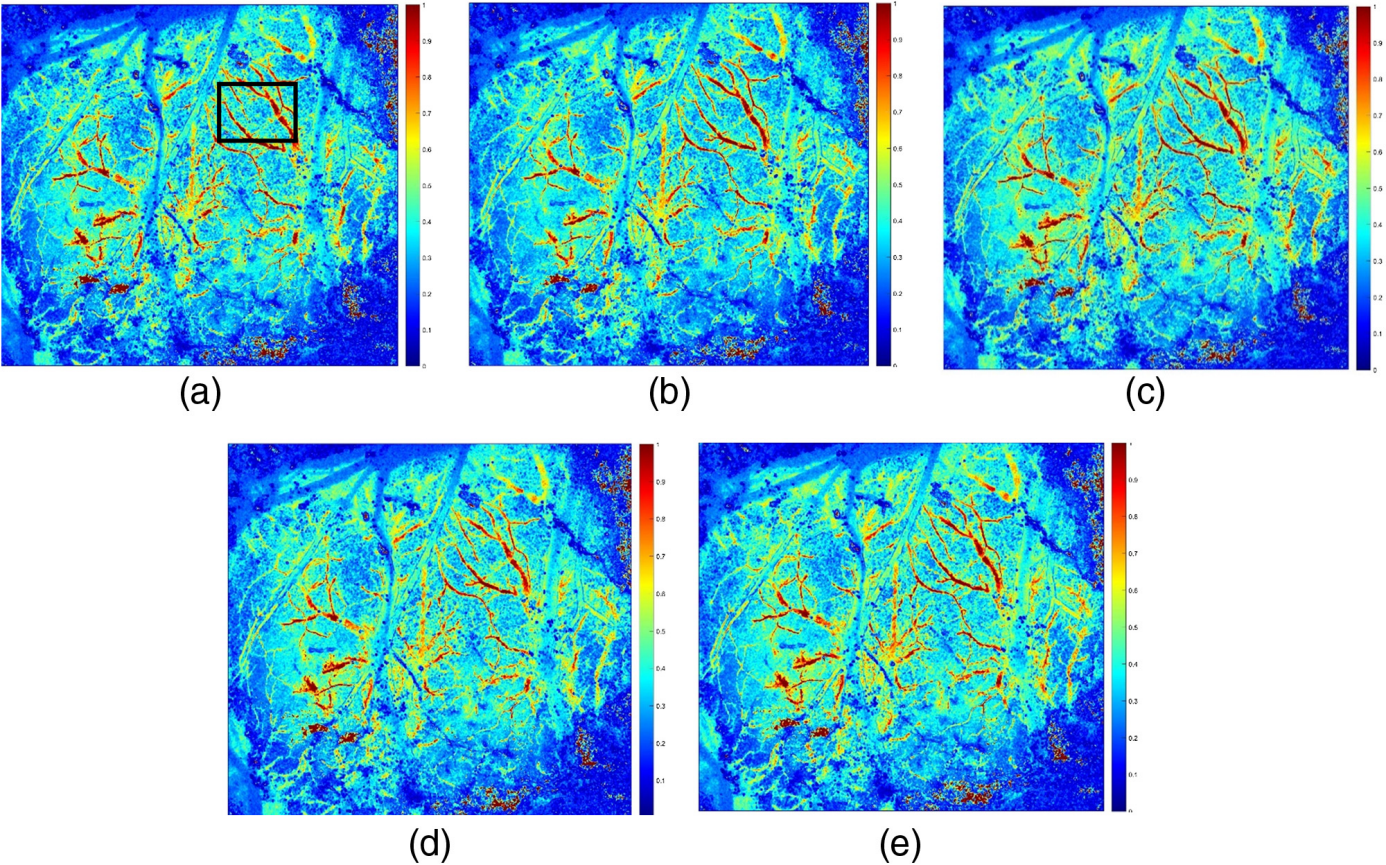
rCBF image of pulse width 3 ns with different repetition rates with 532-nm nanosecond pulse laser source: (a) 1200 kHz, (b) 800 kHz, (c) 300 kHz, (d) 100 kHz, and (e) 10 kHz.

The experiment of rCBF imaging in mice with 532-nm cw and 532-nm nanosecond pulse laser acting alone was carried out. The main parameters of 532-nm nanosecond pulse wavelength laser are pulse width and repetition frequency. The effect of the experiment is more obvious. The pulse width is set to 3 ns. Figure 4 shows a pseudocolor image of rCBF in the mouse under the action of 1200, 800, 300, 100, and 10 kHz repetition frequencies, respectively. At the same time, we performed blood flow imaging experiments with various pulse widths of 5, 7, 10, 15, and 20 ns, respectively. The experimental results showed that the information of rCBF in mice can be obtained effectively using a nanosecond pulse laser, which reflects the development potential of the PAI system in blood flow imaging.

### 3.4 Regional Cerebral Blood Flow Image with 532-nm Nanosecond Pulse Laser Source of Different Pulse Widths

**Fig. 5:**
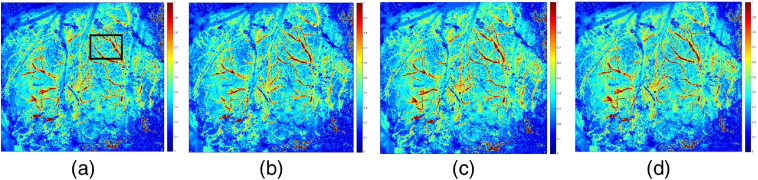
rCBF images of 100 kHz with different pulse widths: (a) 3 ns, (b) 5 ns, (c) 7 ns, and (d) 10 ns.

**Fig. 6:**
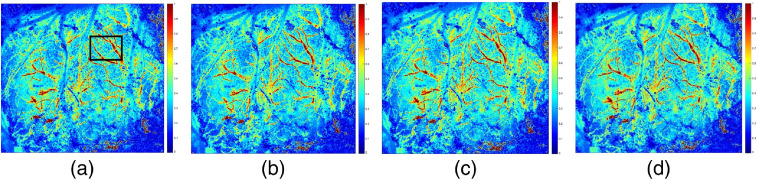
rCBF images of 3 kHz with different pulse widths: (a) 5 ns, (b) 7 ns, (d) 10 ns, and (f) 20 ns.

At the same time, we kept the repetition frequency of the nanosecond pulse laser unchanged and obtained the experimental results of Figs. 5 and 6 by changing the pulse width. The results showed that the change of pulse width had little effect (it can be ignored) on the experimental results of rCBF imaging in mice, and all the results showed similar information of blood vessel and blood flow. After calculating the SNR (refer to Fig. 8), it is found that all the images have the same magnitude range. The experimental results in Figs. 4–6 further showed that the repetition frequency and pulse width of the nanosecond pulse laser have little effect on blood flow imaging in mice.

### 3.5 Calculation Results of Signal-to-Noise Ratio

**Fig. 7:**
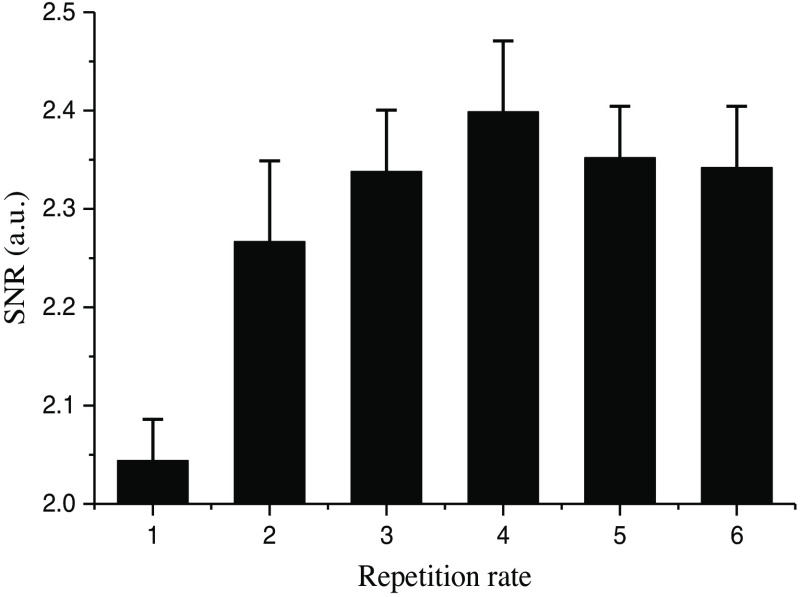
The SNR with 532-nm cw (1) in the ROI and pulse width of 3 ns with a repetition rate of 1200 kHz (2), 800 kHz (3), 300 kHz (4), 100 kHz (5), and 10 kHz (6).

The SNRs of the ROIs of rCBF [see the box lines in Figs. 3(a) and 4] are calculated and shown in Fig. 7. The SNR of the 532-nm cw (SNR=2.04) ROI is significantly smaller than that of 532-nm pulse laser (SNR=2.36, repetition rate of 100 kHz, and pulse width of 3 ns), which demonstrates that the 532-nm nanosecond pulse laser can present a better imaging effect. When the pulse width is 3 ns, the SNR is highest at the repetition frequency of 300 kHz, which indicates that the blood flow imaging is sharper and the noise is less.

**Fig. 8:**
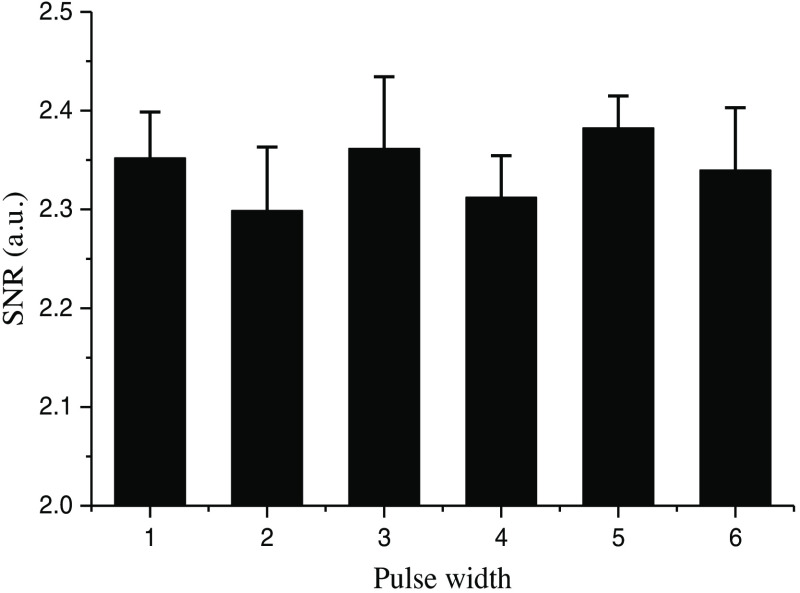
The SNR with the repetition rate of 100 kHz and pulse width of 3 ns (1), 7 ns (2), 10 ns (3), and with a repetition rate of 3 kHz and the pulse width of 7 ns (4), 10 ns (5), and 20 ns (6), respectively.

The ROI in Fig. 3 is selected for the same processing as above, and the resulting SNR is shown in Fig. 8. Under the same repetition frequency, differences in SNR among the images with various pulse widths are very small (SNR is maintained between 2.3 and 2.4), which indicates that different repetition frequencies and pulse widths have little effect on the experimental results of laser speckle blood flow imaging.

## 4 Discussion

Blood supply reflects the functional state of an organism and is an important link to maintain the normal physiological function of an organism.^17^^–^^20^ Oxygen is the basis of life metabolism. Whether or not the oxygen content in the blood is enough is very important to maintain the normal metabolism of a living body. The occurrence of cerebrovascular disease is usually accompanied by the change of rCBF velocity. In the measurement of living biological tissues, especially blood vessels, the commonly used techniques are MRI perfusion imaging, fluorescence angiography imaging, and Doppler blood flow meter. However, in view of the imaging principle of each technique, there are shortcomings in small animal rCBF imaging.^21^^,^^22^ LSCI can provide real-time functional imaging of the whole area without scanning, and the obtained image has many advantages such as high-resolution, fast, noninvasive, and so on.^23^^–^^26^ In this paper, two types of laser sources were used for rCBF imaging in small animals. Considering the defects of the continuous laser source, we have developed a LSCI system composed of a 632.8-nm continuous laser and a 532-nm continuous laser/nanosecond pulse laser. Specifically, the image differences caused by three types of laser sources are compared, including a continuous laser source with the same wavelength as the nanosecond laser source, and a continuous laser source with a different wavelength. The experimental results are different.

The effects of two different wavelengths of 632.8 and 532 nm were shown in Fig. 2. It was found that the image by 532-nm cw laser source showed clear branches of the mouse brain, and the trunk was clear. The effects of two different types of 532 nm were shown in Fig. 3. It was found that 532-nm nanosecond pulse laser exhibits blood flow change capturing more effective blood vessel detailed information. The experimental results, as shown in Figs. 4 and 5, further showed that the repetition frequency and pulse width of the nanosecond pulse laser have little effect on blood flow imaging in mice, which reflects the important research significance of the nanosecond pulse laser for blood flow imaging, and provides the possibility for the development of a multimode PAI system.

Our results show that the 632.8-nm laser is better at detecting main blood vessels at the surface of the mouse brain, while the 532-nm laser is better at capturing small blood vessels in the mouse brain with deeper depth. This may be due to the significant absorption of the 532-nm laser by the main blood vessels. Comparing the imaging results of 532-nm cw and nanosecond pulse laser, the blood flow image of the 532-nm nanosecond pulse laser has higher SNR and lower noise, which proves that the imaging effect is better. Finally, comparing results of the nanosecond pulse laser with various pulse widths at the same repetition frequency and results with various repetition frequencies at the same pulse width, it is confirmed that the repetition frequency and pulse width have little effect on the results of laser speckle blood flow imaging experiments.

There are still several limitations. Only several pulse widths (3, 5, 7, 10, and 20 ns) and several repetition rates (1200, 800, 300, 100, and 10 kHz) were used in the study and the results concerning accuracy would be limited by the number. With the increase of the parameters, this will collect more and more cases and improve the accuracy of rCBF detection. Moreover, we just evaluated one parameter (SNR) and examined the potential efficiency of the feature. To improve the accuracy of diagnosis, additional features could be needed.

The PAI system has been widely used in high-sensitivity structural and blood flow. However, it is time-consuming to obtain a 2-D image of blood flow, which needs multiple repeated A-line imaging at one spot. In this paper, we have shown that a LSCI system can provide 2-D full-speed blood flow images with a nanosecond pulse laser. Thus, the full-speed 2-D blood flow images can be achieved in a PAI system using the imaging method of LSCI.

## 5 Conclusion

In summary, this study confirmed the feasibility of a nanosecond pulse laser for laser speckle blood flow imaging experiments. The 632.8- and 532-nm cw lasers have different imaging characteristics for blood vessels and blood flow in the same brain region, which has certain research value for the fusion of the two images. The rCBF images obtained using a 532-nm nanosecond pulse laser showed higher SNR and better imaging quality than the 532-nm cw laser. In addition, there was no significant difference in results using the nanosecond pulse laser among various pulse widths or repetition rates. This study provides a foundation for subsequent related research and for future multimodal PAI systems and image fusion.
